# Cancer Risk in Men with HIV in Japan: An 18-Year Single-Center Cohort Study

**DOI:** 10.3390/cancers18020248

**Published:** 2026-01-14

**Authors:** Keiji Konishi, Tomoko Uehira, Kazuyuki Hirota, Takashi Ueji, Yasuharu Nishida, Takuma Shirasaka, Dai Watanabe

**Affiliations:** 1 AIDS Medical Center, NHO Osaka National Hospital, Osaka 540-0006, Japan; uehira.tomoko.mz@mail.hosp.go.jp (T.U.); hirota.kazuyuki.wf@mail.hosp.go.jp (K.H.); ueji.takashi.wm@mail.hosp.go.jp (T.U.); nishida.yasuharu.kt@mail.hosp.go.jp (Y.N.); shirasaka.takuma.ca@mail.hosp.go.jp (T.S.); watanabe.dai.pg@mail.hosp.go.jp (D.W.); 2 Department of Post-Infectious Disease Therapeutics, Graduate School of Medicine, The University of Osaka, Osaka 565-0871, Japan; 3 Department of Advanced Medicine for HIV Infection, Graduate School of Medicine, The University of Osaka, Osaka 565-0871, Japan

**Keywords:** HIV, malignant tumor, standardized incidence ratio, AIDS-defining malignancy, non-AIDS-defining malignancy, Japan

## Abstract

**Objectives**: Among people with HIV (PWH), the epidemiology of malignant tumors has shifted from AIDS-defining malignancies (ADMs) to non-AIDS-defining malignancies (NADMs). This study examined temporal changes in the standardized incidence ratio (SIR) of malignant tumors in an HIV cohort in Japan. **Methods**: A retrospective cohort study was conducted of 3793 men treated for HIV at Osaka National Hospital between 2007 and 2024. Diagnoses of malignant tumors were identified from medical records and the expected numbers of cases were calculated using cancer incidence rates for the general male population of Japan. SIRs and 95% confidence intervals (CIs) were calculated and temporal changes across four periods (2007–2011, 2012–2016, 2017–2020, and 2021–2024) were evaluated using the *p* for trend. **Results**: The overall SIR for malignant tumors decreased from 5.12 (95% CI: 4.02–6.43) in 2007–2011 to 0.86 (95% CI: 0.64–1.14) in 2021–2024, mainly owing to a decline in ADMs (SIR: 111.93 to 5.70), including Kaposi’s sarcoma (SIR: 4269.39 to 547.26) and AIDS-related lymphoma (SIR: 62.18 to 3.13). The overall SIR for NADMs was similar to that of the general population (1.04; 95% CI: 0.89–1.22), and decreased from 1.64 to 0.69, but the risks of anal cancer (SIR 40.63) and oral/pharyngeal cancer (SIR 3.16) remained high. **Conclusions**: Among men with HIV in Japan, the overall risk of ADMs and NADMs has decreased; however, the risk of specific NADMs remains high. Cancer prevention strategies for PWH need to focus on high-risk NADMs.

## Simple Summary

Effective treatments allow people with HIV to live longer, but this changes their long-term health risks, including their risk of cancer. We analyzed cancer trends in 3793 men with HIV in Japan over an 18-year period from 2007 to 2024 to understand these changes. We found that the risk of AIDS-defining cancers (Kaposi’s sarcoma and AIDS-related lymphoma) has decreased dramatically. The overall risk of cancers other than the AIDS-defining cancers has also decreased to levels similar to those of the general population. However, the risk for some specific non-AIDS-defining cancers, particularly anal cancer and oral/pharyngeal cancers, remains persistently high. This research shows that cancer prevention strategies for people with HIV in Japan need to be adapted. Healthcare providers should refocus efforts on screening and preventing the specific high-risk cancers to ensure the long-term health of patients with HIV.

## 1. Introduction

The advent and evolution of antiretroviral therapy (ART) have fundamentally transformed the clinical picture of human immunodeficiency virus (HIV) infection. Once a fatal disease, it has become a manageable chronic condition, and the life expectancy of people with HIV (PWH) is now similar to that of the general population [1,2]. In Japan, ART became widely available in the late 1990s. The government provides substantial financial support through the Medical Care for Services and Supports for Persons with Disabilities program, which ensures that out-of-pocket expenses for ART are minimal. This system supports high treatment adherence and long-term viral suppression among Japanese PWH. The success of ART has led to the aging of the PWH population. Along with this shift, the epidemiology of malignant tumors has undergone a transformation. Although the incidence of AIDS-defining malignancies (ADMs) has markedly decreased, the incidence of non-AIDS-defining malignancies (NADMs), which are associated with aging and chronic inflammation, has increased [3,4,5]. A more detailed understanding of this epidemiological transition is essential to develop effective cancer prevention and management strategies for the aging PWH population.

Although the incidence of NADMs has increased worldwide, the changes in risk are not uniform across all cancer types. Marked differences have been reported between “infection-related NADMs,” which are associated with human papillomavirus (HPV) and hepatitis B and C viruses (HBV and HCV), and “non–infection-related NADMs,” which are not associated with infection [6]. Large cohort studies from Europe, North America, and Australia have consistently shown that the risks of infection-related NADMs, such as anal and liver cancers, remain high among PWH, and the incidence has increased in some populations [7]. In contrast, the risks of non-infection-related NADMs, such as lung, colorectal and prostate cancers, are comparable to or even lower than those of the general population. These patterns primarily reflect traditional risk factors in the population, such as aging and the prevalence of smoking [8,9]. However, most previous studies have been conducted in Europe and North America, and long-term detailed analyses of site-specific cancer incidence risk trends among PWH in Japan, in whom the racial background, lifestyle, and cancer epidemiology differ, remain limited [10,11]. This lack of knowledge poses a challenge for the development of country-specific cancer-control strategies.

Based on these considerations, this study investigated temporal changes in site-specific cancer incidence using long-term observational data from a large single-center HIV cohort in Japan. This study attempted to elucidate long-term changes in cancer incidence risk among PWH in Japan and also to confirm, using domestic data, epidemiological transition from ADMs to NADMs internationally.

## 2. Materials and Methods

### 2.1. Study Design

A retrospective cohort study was conducted, covering the 18-year period from 2007 to 2024, and standardized incidence ratios (SIRs) were calculated for overall ADMs, NADMs, and major cancer types. The study period was divided into four time periods, and SIRs were calculated for each period to provide a detailed analysis of the temporal dynamics of the epidemiological profile of cancer among men with HIV in Japan. The study was conducted at Osaka National Hospital. By the end of 2024, the hospital, which serves as one of the country’s major tertiary care centers, had provided care for a cumulative total of 4196 patients with HIV infection, accounting for approximately 12% of all patients with HIV in Japan [12].

### 2.2. Study Population and Period

The study period was from 1 January 2007 to 31 December 2024. The start date of 1 January 2007 was chosen to coincide with the full implementation of the electronic medical record system in our institution. The participants were men who received medical care for HIV at Osaka National Hospital who either visited the hospital for the first time during the study period or had been receiving regular outpatient care before 1 January 2007, and had at least one visit during 2007. Patients who were aged younger than 20 years at their first visit on or after 1 January 2007, and female patients were excluded. Female patients were excluded because they comprised approximately 4% of the cohort, and statistical power for detailed stratified analyses was limited. Participants included both individuals who were newly diagnosed with HIV at our hospital and those who had been diagnosed and followed at other institutions before being referred to our center during the study period. For patients whose first visit to our hospital occurred in or after 2003, we additionally recorded whether HIV had been diagnosed at our hospital or elsewhere and whether the patient had been followed at another facility before referral. These variables were used to describe the cohort and clarify the timing of HIV diagnosis in relation to follow-up initiation.

The study was approved by the Institutional Review Board of Osaka National Hospital (Approval No. 25041) and was conducted in accordance with the principles of the Declaration of Helsinki. Because analyses were performed using anonymized clinical data, the requirement for individual informed consent was waived. Information about the study was disclosed on the hospital’s website, and patients had the option of opting out by refusing to allow the use of their data. No patients opted out during the study period.

### 2.3. Data Sources and Variables

The following variables were collected from medical records: date of the first visit to the hospital, history of AIDS-defining illnesses, details and duration of ART, plasma HIV-1 RNA levels (viral load), CD4 counts, estimated route of HIV infection, date and site of malignant tumor diagnosis (ICD-10 codes), and date of death. In this study, “at registration” refers to the starting point of the follow-up, namely, the date of the first visit to the hospital on or after 1 January 2007. The lowest CD4 count and highest viral load values recorded in each calendar year were used as time-varying measures for that year’s analysis. The follow-up period for each patient started from the date of the first visit to the hospital on or after 1 January 2007, and ended at the earliest of the following: the date of death, the date of the last visit (in cases lost to the follow-up or referred to another institution), or 31 December 2024. Lost to follow-up was defined as the absence of hospital visits for more than 12 months after the last recorded visit, without confirmation of continued care at another medical institution or death. At our hospital, a system is in place whereby a coordinating nurse contacts patients who miss scheduled appointments to encourage reattendance.

Malignant tumors were categorized into ADMs (Kaposi’s sarcoma and AIDS-related non-Hodgkin lymphoma) and NADMs (defined as all malignant tumors other than the specified ADMs). Among them, systemic AIDS-related non-Hodgkin lymphomas included diffuse large B-cell lymphoma, Burkitt lymphoma, and plasmablastic lymphoma [13]. Recurrent malignant tumors at the same site were not counted as separate events, whereas multiple primary cancers occurring at different sites were treated as independent events. To estimate standardized incidence ratios for incident malignancies, cancers diagnosed before the start of follow-up were not counted as study events. Follow-up was initiated at the first HIV clinic visit on/after 1 January 2007 (baseline). Malignancies diagnosed on or before baseline (including cases in which HIV infection was identified during evaluation for cancer or when cancer was detected at the time of HIV diagnosis) were treated as prevalent and excluded from incident-case counting.

### 2.4. Statistical Analysis

The primary outcomes were temporal changes in SIRs for ADMs and NADMs. The SIRs of site-specific NADMs were secondary outcomes. Person-years of observation were calculated for each participant and assigned to each of four predefined calendar periods (2007–2011, 2012–2016, 2017–2020, and 2021–2024) to facilitate the analysis of temporal trends in cancer incidence. These intervals were chosen primarily for statistical convenience to ensure an adequate number of person-years in each group. Age-specific cancer incidence rates among the general male population of Japan were used to estimate the expected number of cases. In each period, age-specific incidence rates from the reference years 2007, 2012, 2017, and 2021 were applied to the corresponding age strata within each period [14]. However, because national age-specific incidence data were unavailable for Kaposi’s sarcoma and anal cancer, estimates were calculated using the overall crude incidence rates for all ages combined. SIRs were obtained by dividing the observed number of cases by the expected number, and 95% confidence intervals (CIs) were calculated assuming a Poisson distribution. SIRs were calculated according to the calendar year, immune status (CD4 count), age group (20–39, 40–64, and ≥65 years), and cancer type. To formally test temporal changes in SIRs, a Poisson regression model was applied to calculate the *p* value for trend (*p*-trend). All statistical analyses were conducted using R software, version 4.5.1 (The R Foundation for Statistical Computing, Vienna, Austria).

## 3. Results

Of the 4196 patients assessed for eligibility, 3793 men with HIV who received treatment at Osaka National Hospital between 2007 and 2024 were included in the analysis (Figure S1).

The clinical characteristics of participants are shown in Table 1. The median follow-up period was 10.1 years (interquartile range [IQR]: 4.1–15.5 years), with a total of 32,818 person-years of observation. At registration, the median age was 36.3 years (IQR: 29.7–44.9 years), the median CD4 count was 274 cells/μL (IQR: 113–420 cells/μL), and the median viral load was 4.7 log10 copies/mL (IQR: 3.8–5.3 log10 copies/mL). A total of 828 patients (21.8%) had a history of AIDS. The most common route of infection was sex between men, accounting for 3122 patients (82.3%), and 3553 participants (93.7%) were of Japanese nationality.

During the study period, 288 malignant tumors were diagnosed (Table 2), of which 127 (44.1%) were ADMs and 161 (55.9%) were NADMs. Of the 127 ADMs, 65 were AIDS-related malignant lymphoma and 62 were Kaposi’s sarcoma. Among the 161 NADMs, the most frequent cancer was gastric cancer (25 cases), followed by lung cancer (21 cases), prostate cancer (17 cases), oral/pharyngeal cancer (16 cases), anal cancer (12 cases), and liver cancer (12 cases). The median CD4 count at the time of ADM diagnosis was 85 cells/μL (IQR: 26–247 cells/μL), whereas the median CD4 count at the time of NADM diagnosis was 411 cells/μL (IQR: 246–533 cells/μL). A total of 97 deaths occurred among patients diagnosed with malignancies, of which malignancy was the primary cause of death in 73 cases.

During the study period, the overall crude incidence rate of all malignant tumors was 865.4 per 100,000 person-years. The temporal changes in the crude incidence rate of all malignant tumors are shown in Table S1. When stratified by period, the crude incidence rate decreased consistently from 1373.63 per 100,000 person-years in 2007–2011 to 536.32 per 100,000 person-years in 2021–2024. This decrease was primarily attributable to a decreased incidence of ADMs. The crude incidence rate of ADMs (the combined total of Kaposi’s sarcoma and malignant lymphoma) decreased from 946.69 per 100,000 person-years in 2007–2011 to 120.40 per 100,000 person-years in 2021–2024. In contrast, the crude incidence rate of NADMs remained stable during the same period, at 426.94 per 100,000 person-years in 2007–2011, and 415.92 per 100,000 person-years in 2021–2024.

**Figure 1:**
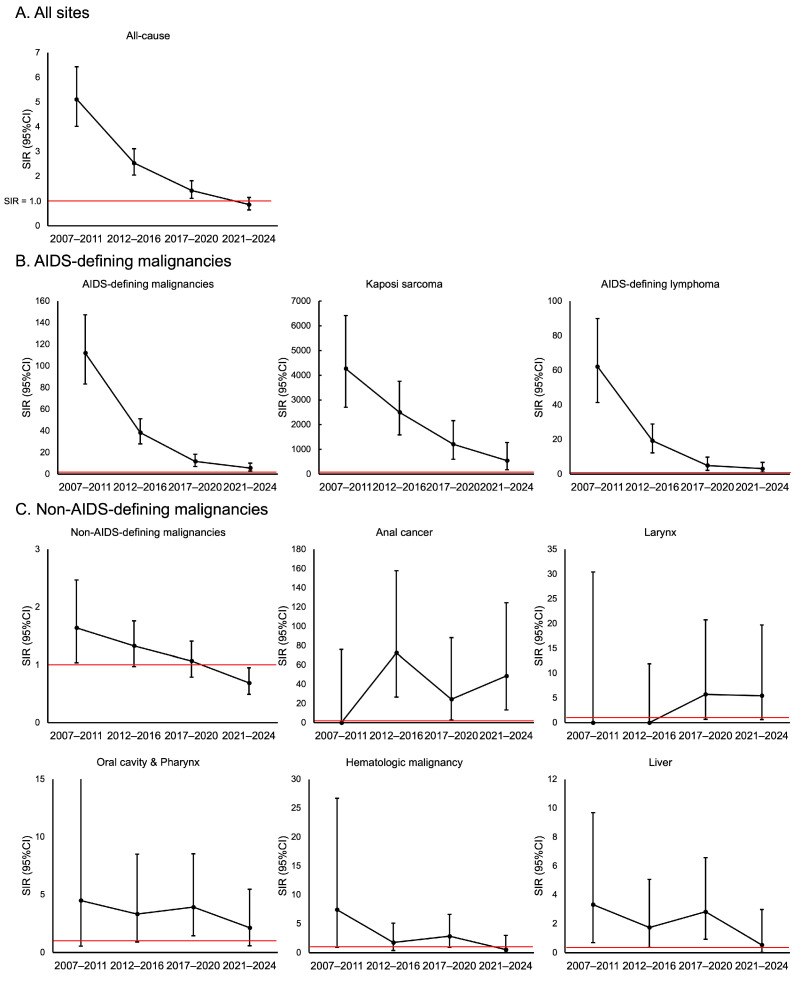
Standardized incidence ratios (SIRs) for malignancies in men with HIV by time period from 2007 to 2024. (**A**) SIRs for all-cause malignancy. (**B**) SIRS for AIDS-defining malignancies (ADMs) (left), including Kaposi sarcoma (middle), and AIDS-defining lymphoma (right), showing substantial decline over the study period. (**C**) SIRS for non-AIDS-defining malignancies (NADMs) (top row, left), including anal cancer (top row, middle), larynx (top row right), oral cavity and pharynx (bottom row, left), hematologic malignancies (bottom row, middle), and liver (bottom row, right). The error bars represent 95% confidence intervals (CIs). Wide 95% CIs for some cancer types reflect limited case numbers during the specific time period. The horizontal line indicates an SIR of 1.0.

The overall risk of all malignant tumors was significantly higher among men with HIV than among men in the general population throughout the study period (SIR 1.83, 95% CI: 1.62–2.05). However, the SIR consistently decreased during the study period. The SIR for all malignant tumors declined significantly from 5.12 (95% CI: 4.02–6.43) in 2007–2011 to 0.86 (95% CI: 0.64–1.14) in 2021–2024 (*p*-trend < 0.001) (Figure 1).

This decrease mainly reflected a marked reduction in ADMs, which declined from an SIR of 111.93 in 2007–2011 to 5.70 in 2021–2024 (*p*-trend < 0.001). The SIR for Kaposi’s sarcoma decreased from 4269.39 in 2007–2011 to 547.26 in 2021–2024 (*p*-trend < 0.001), and that for AIDS-related malignant lymphoma decreased from 62.18 in 2007–2011 to 3.13 in 2021–2024 (*p*-trend < 0.001). Furthermore, the overall SIR for NADMs showed a significant decrease over the same period, from 1.64 in 2007–2011 to 0.69 in 2021–2024 (*p*-trend < 0.001), and the total NADM risk across all periods was similar to that in the general population (SIR 1.04, 95% CI: 0.89–1.22). In contrast to this overall decrease in the SIR for NADMs, the SIRs for some cancer types remained elevated. The SIR for anal cancer was extremely high throughout the study period (40.63, 95% CI: 20.99–70.97), with no significant temporal change (*p*-trend = 0.591). Similarly, the SIR for oral/pharyngeal cancer was persistently higher than that in the general population (SIR 3.16, 95% CI: 1.81–5.14). The risks of other NADMs did not differ significantly from those in the general population. Specifically, the risks of gastric cancer (SIR 1.27, 95% CI: 0.82–1.88), liver cancer (SIR 1.91, 95% CI: 0.99–3.33), and lung cancer (SIR 1.22, 95% CI: 0.76–1.87) were comparable to those in the general population. Rectal cancer was also lower than expected in the overall analysis (SIR 0.38, 95% CI: 0.10–0.97).

When temporal changes in SIRs were examined by age group (Table S2), the relative risk (SIR) for all malignant tumors, ADMs, and NADMs was the highest in the younger age group (20–39 years) and decreased slightly with increasing age. The overall SIRs for all malignant tumors during the study period were 10.78 (95% CI: 8.25–13.85) in the 20–39-year age group, 1.95 (95% CI: 1.67–2.28) in the 40–64-year age group, and 0.90 (95% CI: 0.68–1.15) in the ≥65-year age group. The SIRs for ADMs were considerably higher in the 20–39-year (98.21) and 40–64-year (25.04) age groups than in the ≥65-year age group (4.17). The SIR was significantly higher in the 20–39-year age group (3.45, 95% CI: 2.04–5.45), but the SIRs did not differ significantly from those of the general population in the 40–64-year (1.08) or the ≥65-year (0.81) age groups. The risks of all malignant tumors and ADMs decreased significantly in all age groups over time (*p*-trend < 0.05). The decline in the risk of NADMs was only statistically significant in the 20–39-year age group (*p*-trend = 0.008). In the most recent period (2021–2024), the risk of NADMs among participants aged 40 years or older was slightly lower than that in the general population (40–64 years: SIR 0.72, 95% CI: 0.44–1.11; ≥65 years: SIR 0.69, 95% CI: 0.41–1.09), although these differences were not statistically significant.

The risk of malignant tumors was strongly associated with the immune status at diagnosis (Table 3). The SIR was elevated among participants with CD4 counts <200 cells/μL (8.04, 95% CI: 6.78–9.47). This increased risk was driven primarily by ADMs, with an SIR of 183.33 (95% CI: 148.84–223.42); however, the SIR for NADMs was also significantly higher than that of the general population (2.61, 95% CI: 1.90–3.49). In the group with CD4 counts of 200–499 cells/μL, the SIR for all malignant tumors was lower than that of the general population (1.15, 95% CI: 0.94–1.39), with an SIR of 6.61 (95% CI: 3.98–10.32) for ADMs and 0.97 (95% CI: 0.78–1.20) for NADMs. Among men with CD4 counts ≥500 cells/μL, the overall SIR for all malignant tumors was lower than that of the general population (0.68, 95% CI: 0.46–0.96), and the SIR for NADMs was also lower than that of the general population (0.61, 95% CI: 0.40–0.89), but the SIR for ADMs remained higher than that of the general population (2.54, 95% CI: 0.69–6.49).

## 4. Discussion

This study showed changes in the risk of malignant tumors among men with HIV using large-scale cohort data collected over 18 years at a single institution in Japan. The risk of ADMs decreased markedly with the widespread use of ART and improvement in the immune status; however, it remained higher than that in the general population, particularly among men aged under 65 years. Furthermore, the overall risk of NADMs decreased to a similar level to that in the general population. However, the risks of specific virus-related cancers such as anal and oral/pharyngeal cancers remained elevated. Moreover, the SIR for overall NADMs remained significantly high among men aged 20–39 years. These results illustrate the long-term shift in the epidemiological profile of cancer among PWH in Japan, suggesting that although the overall cancer risk among PWH remains higher than that in the general population, the underlying risk profile has changed in parallel with advances in ART.

The marked decline in the incidence of ADMs during the study period is consistent with the epidemiological changes reported in other HIV cohorts worldwide since the introduction of ART. The marked decreases in SIRs for Kaposi’s sarcoma and AIDS-related malignant lymphoma during the study period confirmed that the temporal changes reported in European and North American cohorts are also evident in Japan. Large collaborative cohort studies in Europe and North America have consistently demonstrated marked reductions in the incidence of Kaposi’s sarcoma and AIDS-related malignant lymphoma following the introduction and widespread use of ART [5,15]. More recent studies including data up to 2019 have also shown continued declines in both the incidence and relative risk of these malignancies among PWH in the United States [16]. In cohorts in Japan, the marked decrease in the SIRs for ADMs from the earlier to later periods of this study confirmed that effective immune reconstitution through ART suppresses the reactivation of oncogenic viruses, such as Epstein–Barr virus (EBV) and human herpesvirus 8, thereby preventing tumor development [5,15,17,18]. A previous study showed that achieving and maintaining viral suppression were directly associated with reductions in the incidence of ADMs [19]. Consistent with findings from other countries, the results of this study suggest that the risk of AIDS-related malignant lymphoma, although markedly reduced, remains higher than that in the general population. These findings suggest that even mild immunodeficiency is a risk factor for AIDS-related malignant lymphoma [7,20]. The results highlight the importance of maintenance of immune competence through continuous ART.

The finding that the overall risk of NADMs was similar to that in the general population and decreased significantly during the study period is an indication of the overall success of cancer management among PWH in Japan. The finding that the overall risk of NADMs was similar to that in the general population is consistent with the results of a study from South Korea [21]; however, other studies have reported higher risks of NADMs among men with HIV than those in the general population [22], suggesting that regional differences and differences in study design need to be considered. Importantly, this overall risk level conceals considerable heterogeneity in risk by cancer type, as well as marked differences in relative risk by age group. Among younger individuals aged 20–39 years, the overall SIR for NADMs remained significantly elevated, indicating that despite advances in the ART era, the risk in this age group remains elevated. The temporal decline in the risk of NADMs was significant only in the 20–39-year age group. Although the risk of NADMs did not increase significantly among those aged 40 years and older, in recent years, it has not exceeded that of the general population.

By taking these age-related differences in risk into consideration, the site-specific analysis revealed that the risk of anal cancer was extremely high and showed no significant decrease throughout the study period. This extremely high risk is consistent with findings from HIV cohorts worldwide, including those in North America [5,16] and South Korea [21,22], and has been attributed to the high prevalence of persistent HPV infection among men who have sex with men (MSM) [23]. The persistently high risk of anal cancer, despite the decreasing risks of ADMs and many other NADMs, indicates that the prevention and management of anal cancer remains an important issue among PWH in Japan [23].

Similarly, the risk of oral/pharyngeal cancer was significantly elevated, consistent with findings among U.S. veterans [24] and Korean men [22]. The persistently high risk of oral/pharyngeal cancer may be attributed to multiple factors, including HPV infection, smoking, and alcohol consumption [24].

In contrast to the findings of some other studies, this study did not detect significant increases in the risks of liver cancer, gastric cancer, or lung cancer compared with those in the general population. This result contrasts with the findings of a study conducted in the United States that showed persistently elevated risks of liver and lung cancers among PWH [16]. The increased risk of liver cancer was attributed to a high prevalence of HBV/HCV coinfection [25]. In this study, the SIR for liver cancer was not significantly elevated compared with that in the general population. In the earlier periods of this cohort, hepatocellular carcinoma was more frequently observed among patients with hemophilia who had acquired HIV through contaminated blood products, many of whom were coinfected with HCV [26,27]. A multicenter study in Japan reported that HCV-related hepatocellular carcinoma is among the most significant problems in this population, with the cause of death shifting from AIDS-related diseases to HCV-related cirrhosis and hepatocellular carcinoma around 2010 [27]. However, the contribution of HCV-related hepatocellular carcinoma has decreased over time owing to successful treatment with direct-acting antivirals. A recent study reported that the HCV antibody positivity rate among HIV-infected men who have sex with men in Japan was approximately 3.2–4%, and high rates of sustained virologic response have been achieved with direct-acting antiviral therapy [28,29]. In contrast, HBV coinfection has contributed minimally to hepatocellular carcinoma development in this cohort. These advances in viral hepatitis management have contributed to the declining trend in liver cancer risk among PWH in Japan.

However, studies from South Korea reported only limited increases in the risk of liver, stomach, and lung cancers [21,22]. A Japanese study [11] previously identified these cancers as among the most frequent NADMs; however, the risk of these cancers relative to that of the general population may not necessarily be high. Studies from Japan and South Korea, the main countries in Asia in which gastric cancer is prevalent, suggest a higher risk of gastric cancer among PWH [10,30,31]. A high background prevalence of *Helicobacter pylori* infection has been suggested to interact with HIV-related immunodeficiency, thereby increasing the risk of gastric cancer. However, this study found no significant increase in the risk of gastric cancer, suggesting that the widespread implementation of *H. pylori* eradication therapy and the improved immune status with ART have contributed to a reduction in risk.

Furthermore, the significantly lower risk of rectal cancer observed in this study is consistent with findings from large cohort studies, which show lower rates of colorectal cancer among PWH [16,21,32]. A study using data from the HIV/AIDS Cancer Match Study in the United States reported that the SIR for rectal cancer among PWH was 0.69, and this inverse association applied to all tumor stages, including advanced tumors that are not generally detected on screening [32]. This suggests that differential screening alone cannot fully explain the reduced risk of rectal cancer, and that biological factors may also have contributed to this finding. Notably, HIV infection is known to cause disruption of the gut’s mucosal integrity and alterations in gut microbiota composition, leading to chronic immune activation [33]. These alterations may paradoxically affect colorectal carcinogenesis through mechanisms that remain to be fully elucidated. Further research is needed to clarify the underlying mechanisms for this observation.

In this study, the overall risk of NADMs among PWH aged 40 years and older was similar to that of the general population, which may, in part, reflect the effects of good medical access. Furthermore, the correlation between the CD4 count and risk of malignant tumors observed in this study highlights the central role of CD4 T-cells in tumor immune surveillance. Case reports have also demonstrated that immunodeficiency is a key factor in the development of HIV-associated lymphoma [34]. The results showing that the SIR for all malignant tumors was significantly elevated among patients with CD4 counts <200 cells/μL, whereas the SIR for NADMs was lower than that in the general population among those with CD4 counts ≥500 cells/μL, are particularly important. Matsui et al. [35] experimentally demonstrated that CD4 T-cells regulate the functional “quality” of CD8-positive cytotoxic T lymphocytes (CTLs) and affect the integrity of tumor immune surveillance. The depletion of CD4 T-cells caused by HIV infection disrupts this regulatory mechanism and promotes immune evasion by tumor cells. Furthermore, a longitudinal study by Piriou et al. [36] reported that PWH showed a marked decline in EBV nuclear antigen 1 (EBNA1)-specific CD4 and CD8 T-cells prior to the onset of AIDS-related non-Hodgkin lymphoma, suggesting that the loss of virus-specific T-cell responses is a predictor of tumor development. In this study, the median CD4 count at the time of ADM diagnosis was 85 cells/μL, which supports the hypothesis of virus reactivation-induced oncogenesis under conditions of severe immunodeficiency.

The results of this study provide important insights for the direction of cancer-control strategies among PWH in Japan. Although the overall risk of NADMs has remained at a similar level to that in the general population, the persistently elevated risks of specific cancers, such as anal and oral/pharyngeal cancers, indicate that individualized, risk-based approaches to prevention and early detection rather than uniform strategies are essential.

In particular, for anal cancer, recent evidence has demonstrated the effectiveness of treating high-grade squamous intraepithelial lesions for the prevention of cancer [37]. In response, the International Anal Neoplasia Society published consensus guidelines recommending screening for high-risk groups, including MSM and PWH [38]. These guidelines specify the starting age for screening according to the risk level (e.g., 35 years for HIV-positive MSM and transgender women, and 45 years for other HIV-positive individuals) and recommend specific screening and management methods, such as cytology and HPV testing. The results of this study strongly support the need to introduce and optimize these international recommendations for anal cancer, which remains prevalent among MSM, in Japan.

Similarly, for oral/pharyngeal cancer, strengthening smoking cessation programs and implementing targeted clinical examinations, such as visual and palpation-based screenings, may become increasingly important. In advancing measures against these high-risk NADMs, reinforcing primary prevention efforts, including promoting HPV vaccination and supporting smoking cessation, are essential. In Japan, the active recommendation of HPV vaccination was suspended for approximately 9 years from 2013 to 2022, resulting in a marked decline in vaccination coverage, and the effects of this suspension still persist [39,40]. Moreover, routine HPV vaccination for males has not yet been introduced in Japan, and measures to prevent HPV infection among MSM remain inadequate.

In addition, the implementation of anal cancer screening faces infrastructural challenges, such as the need to expand facilities and train specialists capable of performing high-resolution anoscopy. Furthermore, several U.S. cohort studies have reported low implementation rates of recommended cancer screenings, such as anal cytology, and breast, colorectal, liver, and lung cancer screening [41], and similar issues regarding the uptake and dissemination of screening may also arise in Japan. These findings suggest that future HIV care must evolve beyond viral suppression alone toward a more comprehensive healthcare management model that incorporates the prevention and early detection of comorbidities, including cancer. The results of this study not only support the need to introduce and adapt international recommendations to Japan’s clinical context but also highlight the necessity of developing risk-stratified screening strategies based on cost-effectiveness analyses [42]. Because the epidemiological profile of cancer among PWH has shifted markedly, with NADMs now predominating, the scope of HIV care must be extended beyond viral suppression to encompass the integrated management of comorbidities, including malignancies [43].

This study has several limitations. Because it was conducted at a single institution, the generalizability of the results to the population of PWH of Japan may be limited. Because more than 95.0% of PWH with newly diagnosed HIV infection in Japan are male [12], the focus on cancer incidence among men with HIV in this study aligns with a key national public health priority and does not markedly compromise its generalizability. The results may not reflect regional variations or disparities in healthcare access. Furthermore, because female patients were excluded, it was not possible to examine potential sex-based differences in the incidence of cancer among PWH. Although this exclusion was because of limitations in statistical power, a more detailed understanding of the dynamics of HIV-associated cancer risks among women is an important topic for future research. Detailed information on key confounding factors, such as smoking history, alcohol consumption, and comorbidities, was also limited and, thus, residual confounding due to these factors cannot be ruled out. Furthermore, information on tumor stage at diagnosis was not available in the medical records, which precluded analysis of stage-specific cancer incidence and limited our ability to assess whether HIV status was associated with more advanced disease at presentation. Additionally, the study period overlapped with the COVID-19 pandemic. Potential delays in the diagnosis of both HIV infection and malignancies due to disruptions in healthcare access during this period may have influenced the incidence estimates in the most recent period (2021–2024). Another limitation is that although age-specific incidence rates in the general population were used to calculate the expected number of cases, representative reference years were applied for each study period rather than continuous annual data. Moreover, because nationwide age-specific data were unavailable for Kaposi’s sarcoma and anal cancer, the estimates were based on crude incidence rates. The lack of cross-validation with national cancer registry data may also have led to missed cases diagnosed at other institutions. Additionally, although further analyses by age group were performed, the limited number of cases prevented a detailed age-stratified analysis of site-specific cancer risks.

Future studies need to establish large-scale, multicenter collaborative datasets, include analyses by sex, collect detailed information on lifestyle factors, and improve case ascertainment through integration with national cancer registries. Furthermore, evaluating the effectiveness and cost-effectiveness of screening strategies for high-risk NADMs, particularly anal cancer screening, is an urgent research priority in Japan.

## 5. Conclusions

In conclusion, to the best of our knowledge, this is the first study to demonstrate temporal changes in the standardized incidence ratio of malignant tumors among men with HIV in Japan. This study demonstrates that the epidemiology of cancer among men with HIV in Japan has entered a new phase characterized by a marked decline in ADMs and the persistence of some virus-related NADMs as a continuing challenge. This epidemiological transition suggests that the establishment and implementation of individualized cancer prevention and early detection programs will be crucial for improving long-term survival among PWH.

## Figures and Tables

**Table 1:** Characteristics of study participants (N = 3793).

Variable	Value
Total duration of follow-up for the whole cohort (person-years)	32,818
Age at enrollment (years), median (IQR)	36.3 (29.7–44.9)
Japanese nationality, *n* (%)	3553 (93.7)
CD4 count at enrollment (cells/μL), median (IQR)	274 (113–420)
HIV RNA level at enrollment (log_10_ copies/mL), median (IQR)	4.7 (3.8–5.3)
History of AIDS-defining illness, *n* (%)	828 (21.8)
Route of HIV transmission, *n* (%)	
Male-to-male sexual contact	3122 (82.3)
Heterosexual contact	439 (11.6)
Contaminated blood products	69 (1.8)
Injection drug use	5 (0.1)
Other/Unknown	158 (4.2)
Length of follow-up (years), median (IQR)	10.1 (4.1–15.5)

**Table 2:** Incidence of AIDS-defining and non-AIDS-defining malignancies.

Malignancy	Number of Cases
AIDS-defining malignancies	127
AIDS-defining lymphoma	65
Kaposi’s sarcoma	62
Non-AIDS-defining malignancies	161
Stomach	25
Lung	21
Prostate	17
Oral cavity/pharynx	16
Anal cancer	12
Liver	12
Colon	9
Non-AIDS-defining lymphoma	8
Hematologic malignancy	6
Bladder	5
Skin	5
Larynx	4
Rectum	4
Kidney/urinary tract (excluding bladder)	4
Esophagus	4
Testicular cancer	3
Gallbladder/bile duct	2
Pancreas	2
Brain/central nervous system	1
Adrenal cancer	1

**Table 3:** Crude incidence rate and standardized incidence rate of malignancies according to the CD4 count.

	CD4+ Count (Cells/μL)		
Malignancy type	<200	200–499	≥500
All malignancies			
Crude incidence rate (per 100,000 PY)	4794.1	572.9	269
SIR (95% CI)	8.04 (6.78–9.47)	1.15 (0.94–1.39)	0.68 (0.46–0.96)
AIDS-defining malignancies			
Crude incidence rate (per 100,000 PY)	3285.4	104.7	34.7
SIR (95% CI)	183.33 (148.84–223.42)	6.61 (3.98–10.32)	2.54 (0.69–6.49)
Non-AIDS-defining malignancies			
Crude incidence rate (per 100,000 PY)	1508.6	468.3	234.3
SIR (95% CI)	2.61 (1.90–3.49)	0.97 (0.78–1.20)	0.61 (0.40–0.89)

CI, confidence interval; PY, person-years; SIR, standardized incidence ratio.

## Data Availability

The data presented in this study are not publicly available due to legal and ethical restrictions.

## Supplementary Materials

The following supporting information can be downloaded at: https://www.mdpi.com/article/10.3390/cancers18020248/s1, Figure S1: Flow chart showing participant selection; Table S1: Crude incidence rates and standardized incidence ratios of major malignancies by time period; Table S2: Crude incidence rates and standardized incidence ratios for all malignancies, AIDS-defining malignancies, and non-AIDS-defining malignancies stratified by age group and time period.

## Abbreviations

The following abbreviations are used in this manuscript:

ADM	AIDS-defining malignancy
ART	Antiretroviral therapy
AIDS	Acquired immunodeficiency syndrome
CI	Confidence interval
CTL	Cytotoxic T lymphocyte
EBNA1	Epstein–Barr virus nuclear antigen 1
EBV	Epstein–Barr virus
HBV	Hepatitis B virus
HCV	Hepatitis C virus
HIV	Human immunodeficiency virus
HPV	Human papillomavirus
MSM	Men who have sex with men
NADM	Non-AIDS-defining malignancy
PWH	People with HIV
SIR	Standardized incidence ratio
