# Supplementary material for: Cancer Risk in Men with HIV in Japan: An 18-Year Single-Center Cohort Study
Source: Cancers (Basel). 2026 Jan 14;18(2):248. doi: 10.3390/cancers18020248 (PMC12839040; doi:10.3390/cancers18020248)
Supplement: Supplementary file 1 [file cancers-18-00248-s001.zip › cancers-4075534-supplementary.pdf]

cancers MDPI

# Supplementary Material: Cancer Risk in Japanese Men with HIV: An 18-year Single-Center Cohort Study

**Keiji Konishi, Tomoko Uehira, Kazuyuki Hirota, Takashi Ueji, Yasuharu Nishida, Takuma Shirasaka and Dai Watanabe**

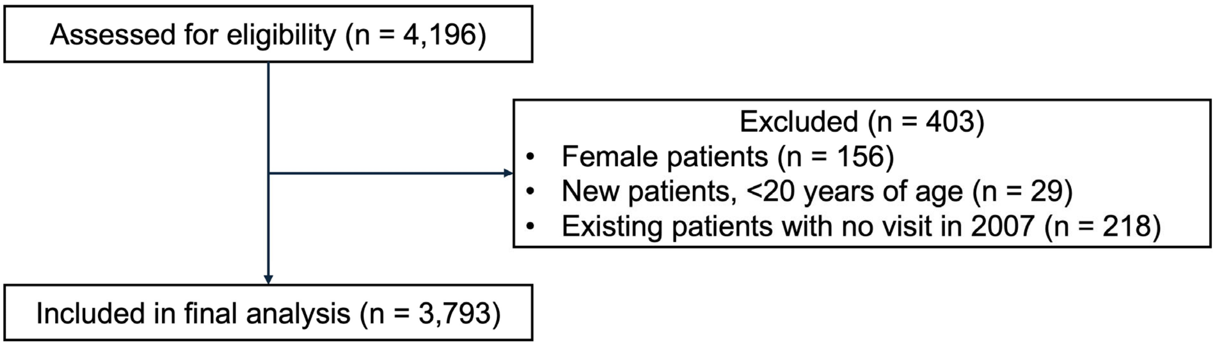


**Figure S1.** Flow chart showing participant selection.

**Table S1.** Crude incidence rates and standardized incidence ratios of major malignancies by time period.

| | **Entire period** | **2007–2011** | **2012–2016** | **2017–2020** | **2021–2024** | ***p*-trend** |
|---|---|---|---|---|---|---|
| All sites | | | | | | |
| Crude incidence rate (/100,000 PY) | 865.4 | 1373.6 | 1011.5 | 747.2 | 536.3 | |
| SIR (95% CI) | 1.83 (1.62–2.05) | 5.12 (4.02–6.43) | 2.54 (2.05–3.11) | 1.43 (1.11–1.82) | 0.86 (0.64–1.14) | <0.001 |
| AIDS-defining malignancies | | | | | | |
| Crude incidence rate (/100,000 PY) | 387.0 | 946.7 | 500.3 | 208.8 | 120.4 | |
| SIR (95% CI) | 24.46 (20.39–29.10) | 111.9 (83.34–147.2) | 38.35 (28.08–51.15) | 11.81 (7.11–18.44) | 5.70 (2.85–10.21) | <0.001 |
| AIDS-related lymphoma | | | | | | |
| Crude incidence rate (/100,000 PY) | 198.1 | 519.8 | 250.2 | 87.91 | 65.67 | |
| SIR (95% CI) | 12.60 (9.72–16.06) | 62.18 (41.32–89.87) | 19.32 (12.25–28.99) | 5.00 (2.16–9.85) | 3.13 (1.15–6.80) | <0.001 |
| Kaposi's sarcoma | | | | | | |
| Crude incidence rate (/100,000 PY) | 188.9 | 426.9 | 250.2 | 120.9 | 54.73 | |
| SIR (95% CI) | 1889 (1448–2422) | 4269 (2706–6406) | 2502 (1586–3754) | 1209 (603.4–2163) | 547.3 (177.7–1277) | <0.001 |
| Non-AIDS-defining malignancies | | | | | | |
| Crude incidence rate (/100,000 PY) | 478.39 | 426.94 | 511.19 | 538.42 | 415.92 | |
| SIR (95% CI) | 1.04 (0.89–1.22) | 1.64 (1.04–2.47) | 1.33 (0.97–1.76) | 1.07 (0.79–1.41) | 0.69 (0.49–0.95) | <0.001 |
| Anal cancer | | | | | | |
| Crude incidence rate (/100,000 PY) | 36.56 | 0 | 65.26 | 21.98 | 43.78 | |
| SIR (95% CI) | 40.63 (20.99–70.97) | 0.00 (0.00–76.08) | 72.51 (26.61–157.8) | 24.42 (2.96–88.21) | 48.65 (13.25–124.6) | 0.591 |
| Larynx | | | | | | |
| Crude incidence rate (/100,000 PY) | 12.19 | 0 | 0 | 21.98 | 21.89 | |
| SIR (95% CI) | 3.49 (0.95–8.94) | 0.00 (0.00–30.45) | 0.00 (0.00–11.92) | 5.74 (0.70–20.75) | 5.46 (0.66–19.74) | 0.211 |
| Oral cavity & pharynx | | | | | | |
| Crude incidence rate (/100,000 PY) | 48.75 | 37.13 | 43.51 | 65.93 | 43.78 | |
| SIR (95% CI) | 3.16 (1.81–5.14) | 4.49 (0.54–16.21) | 3.32 (0.91–8.51) | 3.92 (1.44–8.53) | 2.13 (0.58–5.46) | 0.401 |

| | Entire period | 2007–2011 | 2012–2016 | 2017–2020 | 2021–2024 | *p*-trend |
|---|---|---|---|---|---|---|
| Hematologic malignancy | | | | | | |
| Crude incidence rate (/100,000 PY) | 18.28 | 37.13 | 21.75 | 21.98 | 0 | |
| SIR (95% CI) | 2.24 (0.82–4.88) | 7.40 (0.90–26.73) | 3.10 (0.37–11.18) | 2.50 (0.30–9.03) | 0.00 (0.00–3.85) | 0.038 |
| Skin | | | | | | |
| Crude incidence rate (/100,000 PY) | 15.24 | 18.56 | 10.88 | 10.99 | 21.89 | |
| SIR (95% CI) | 2.03 (0.66–4.73) | 5.66 (0.14–31.52) | 1.93 (0.05–10.77) | 1.23 (0.03–6.88) | 2.08 (0.25–7.51) | 0.575 |
| Non-AIDS-related malignant lymphoma | | | | | | |
| Crude incidence rate (/100,000 PY) | 24.38 | 37.13 | 21.75 | 43.95 | 0 | |
| SIR (95% CI) | 1.93 (0.83–3.81) | 4.44 (0.54–16.05) | 1.68 (0.20–6.07) | 2.50 (0.68–6.40) | 0.00 (0.00–1.92) | 0.048 |
| Liver | | | | | | |
| Crude incidence rate (/100,000 PY) | 36.56 | 55.69 | 32.63 | 54.94 | 10.95 | |
| SIR (95% CI) | 1.91 (0.99–3.33) | 3.31 (0.68–9.68) | 1.73 (0.36–5.06) | 2.82 (0.91–6.57) | 0.53 (0.01–2.97) | 0.187 |
| Bladder | | | | | | |
| Crude incidence rate (/100,000 PY) | 15.24 | 37.13 | 10.88 | 21.98 | 0 | |
| SIR (95% CI) | 1.47 (0.48–3.43) | 6.13 (0.74–22.15) | 1.33 (0.03–7.41) | 1.96 (0.24–7.09) | 0.00 (0.00–2.84) | 0.046 |
| Stomach | | | | | | |
| Crude incidence rate (/100,000 PY) | 76.18 | 74.25 | 97.89 | 54.94 | 76.62 | |
| SIR (95% CI) | 1.27 (0.82–1.88) | 1.65 (0.45–4.22) | 1.65 (0.75–3.12) | 0.86 (0.28–2.00) | 1.18 (0.47–2.42) | 0.38 |
| Lung | | | | | | |
| Crude incidence rate (/100,000 PY) | 63.99 | 74.25 | 65.26 | 76.92 | 43.78 | |
| SIR (95% CI) | 1.22 (0.76–1.87) | 2.52 (0.69–6.45) | 1.48 (0.54–3.23) | 1.35 (0.54–2.77) | 0.63 (0.17–1.61) | 0.052 |
| Prostate | | | | | | |
| Crude incidence rate (/100,000 PY) | 51.8 | 0 | 43.51 | 65.93 | 76.62 | |
| SIR (95% CI) | 1.04 (0.61–1.67) | 0.00 (0.00–4.05) | 1.18 (0.32–3.03) | 1.18 (0.43–2.58) | 1.01 (0.41–2.09) | 0.749 |
| Brain & central nervous system (CNS) | | | | | | |
| Crude incidence rate (/100,000 PY) | 3.05 | 0 | 0 | 0 | 10.95 | |
| SIR (95% CI) | 0.83 (0.02–4.61) | 0.00 (0.00–24.72) | 0.00 (0.00–12.70) | 0.00 (0.00–9.61) | 2.59 (0.07–14.45) | >0.999 |
| Gallbladder & bile duct | | | | | | |
| Crude incidence rate (/100,000 PY) | 6.09 | 18.56 | 0 | 10.99 | 0 | |
| SIR (95% CI) | 0.96 (0.12–3.48) | 4.80 (0.12–26.73) | 0.00 (0.00–7.16) | 1.60 (0.04–8.90) | 0.00 (0.00–5.07) | 0.235 |
| Esophagus | | | | | | |
| Crude incidence rate (/100,000 PY) | 12.19 | 0 | 43.51 | 0 | 0 | |
| SIR (95% CI) | 0.74 (0.20–1.90) | 0.00 (0.00–6.77) | 3.22 (0.88–8.26) | 0.00 (0.00–2.31) | 0.00 (0.00–1.85) | 0.085 |
| Colon | | | | | | |
| Crude incidence rate (/100,000 PY) | 27.42 | 37.13 | 32.63 | 32.96 | 10.95 | |
| SIR (95% CI) | 0.68 (0.31–1.29) | 1.69 (0.20–6.10) | 1.00 (0.21–2.93) | 0.73 (0.15–2.13) | 0.20 (0.01–1.12) | 0.06 |
| Kidney & urinary tract (excluding bladder) | | | | | | |
| Crude incidence rate (/100,000 PY) | 12.19 | 0 | 21.75 | 0 | 21.89 | |
| SIR (95% CI) | 0.67 (0.18–1.72) | 0.00 (0.00–7.92) | 1.54 (0.19–5.58) | 0.00 (0.00–1.93) | 0.88 (0.11–3.17) | 0.986 |
| Pancreas | | | | | | |
| Crude incidence rate (/100,000 PY) | 6.09 | 0 | 0 | 10.99 | 10.95 | |
| SIR (95% CI) | 0.39 (0.05–1.41) | 0.00 (0.00–8.30) | 0.00 (0.00–3.29) | 0.65 (0.02–3.61) | 0.49 (0.01–2.74) | 0.491 |
| Rectum | | | | | | |
| Crude incidence rate (/100,000 PY) | 12.19 | 0 | 0 | 21.98 | 21.89 | |
| SIR (95% CI) | 0.38 (0.10–0.97) | 0.00 (0.00–3.87) | 0.00 (0.00–1.48) | 0.62 (0.08–2.25) | 0.51 (0.06–1.84) | 0.29 |

CI, confidence interval; PY, person-years; SIR, standardized incidence ratio.

**Table S2.** Crude incidence rates and standardized incidence ratios for all malignancies, AIDS-defining malignancies, and non-AIDS-defining malignancies, stratified by age group and time period.

| | **Entire period** | **2007–2011** | **2012–2016** | **2017–2020** | **2021–2024** | ***p*-trend** |
|---|---|---|---|---|---|---|
| **All sites** | | | | | | |
| Age group: 20–39 years | | | | | | |
| Crude incidence rate (/100,000 PY) | 552.79 | 958.90 | 732.74 | 229.18 | 97.43 | |
| SIR (95% CI) | 10.78 (8.25–13.85) | 22.15 (14.60–32.22) | 14.78 (9.66–21.66) | 4.09 (1.50–8.91) | 1.65 (0.20–5.96) | <0.001 |
| Age group: 40–64 years | | | | | | |
| Crude incidence rate (/100,000 PY) | 843.68 | 1669.58 | 988.03 | 785.89 | 454.91 | |
| SIR (95% CI) | 1.95 (1.67–2.28) | 4.40 (3.14–5.99) | 2.36 (1.75–3.10) | 1.85 (1.35–2.48) | 0.97 (0.64–1.40) | <0.001 |
| Age group: ≥65 years | | | | | | |
| Crude incidence rate (/100,000 PY) | 2532.30 | 4123.79 | 3339.35 | 2259.12 | 2046.29 | |
| SIR (95% CI) | 0.90 (0.68–1.15) | 1.69 (0.68–3.49) | 1.21 (0.69–1.96) | 0.78 (0.46–1.26) | 0.71 (0.43–1.11) | 0.027 |
| **AIDS-defining malignancies** | | | | | | |
| Age group: 20–39 years | | | | | | |
| Crude incidence rate (/100,000 PY) | 389.67 | 745.81 | 450.91 | 152.78 | 97.43 | |
| SIR (95% CI) | 98.21 (71.07–132.28) | 223.69 (138.47–341.93) | 128.77 (73.61–209.12) | 31.74 (8.65–81.27) | 21.34 (2.58–77.10) | <0.001 |
| Age group: 40–64 years | | | | | | |
| Crude incidence rate (/100,000 PY) | 396.12 | 1126.97 | 542.45 | 244.50 | 129.97 | |
| SIR (95% CI) | 25.04 (19.76–31.30) | 94.77 (62.45–137.89) | 36.56 (24.30–52.84) | 14.99 (8.19–25.15) | 7.34 (3.17–14.46) | <0.001 |
| Age group: ≥65 years | | | | | | |
| Crude incidence rate (/100,000 PY) | 300.44 | 1767.34 | 417.42 | 132.89 | 107.70 | |
| SIR (95% CI) | 4.17 (1.68–8.58) | 39.07 (8.06–114.17) | 6.46 (0.78–23.35) | 1.82 (0.05–10.14) | 1.34 (0.03–7.48) | 0.002 |
| **Non-AIDS-defining malignancies** | | | | | | |
| Age group: 20–39 years | | | | | | |
| Crude incidence rate (/100,000 PY) | 163.12 | 213.09 | 281.82 | 76.39 | 0.00 | |
| SIR (95% CI) | 3.45 (2.04–5.45) | 5.33 (1.96–11.61) | 6.12 (2.93–11.25) | 1.49 (0.18–5.39) | 0.00 (0.00–3.30) | 0.008 |
| Age group: 40–64 years | | | | | | |
| Crude incidence rate (/100,000 PY) | 447.56 | 542.61 | 445.58 | 541.39 | 324.93 | |
| SIR (95% CI) | 1.08 (0.86–1.33) | 1.48 (0.79–2.53) | 1.10 (0.70–1.65) | 1.33 (0.90–1.88) | 0.72 (0.44–1.11) | 0.064 |
| Age group: ≥65 years | | | | | | |
| Crude incidence rate (/100,000 PY) | 2231.86 | 2356.45 | 2921.93 | 2126.24 | 1938.59 | |
| SIR (95% CI) | 0.81 (0.60–1.06) | 0.99 (0.27–2.52) | 1.08 (0.59–1.82) | 0.76 (0.43–1.23) | 0.69 (0.41–1.09) | 0.226 |

CI, confidence interval; PY, person-years; SIR, standardized incidence ratio.
